# Mobility enhancement for high stability tungsten-doped indium-zinc oxide thin film transistors with a channel passivation layer

**DOI:** 10.1039/c7ra13193c

**Published:** 2018-02-12

**Authors:** Dun-Bao Ruan, Po-Tsun Liu, Yu-Chuan Chiu, Po-Yi Kuo, Min-Chin Yu, Kai-jhih Gan, Ta-Chun Chien, Simon M. Sze

**Affiliations:** Department of Electronics Engineering and Institute of Electronics, National Chiao Tung University Hsinchu 30010 Taiwan; Department of Photonics and Institute of Electro-Optical Engineering, National Chiao Tung University Hsinchu 30010 Taiwan ptliu@mail.nctu.edu.tw +886-3-5712121-52994

## Abstract

This study investigates the electrical characteristics and physical analysis for an amorphous tungsten-doped indium-zinc oxide thin film transistor with different backchannel passivation layers (BPLs), which were deposited by an ion bombardment-free process. A 10 times increase in mobility was observed and attributed to the generation of donor-like oxygen vacancies at the backchannel, which is induced by the oxygen desorption and Gibbs free energy of the BPL material. The mechanism was well studied by XPS analysis. On the other hand, a HfO_2_ gate insulator was applied for the InWZnO TFT device to control the extremely conductive channel and adjust the negative threshold voltage. With both a HfO_2_ gate insulator and a suitable BPL, the InWZnO TFT device exhibits good electrical characteristics and a remarkable lifetime when exposed to the ambient air.

## Abstract 2

This study investigates the electrical characteristics and physical analysis for an amorphous tungsten-doped indium-zinc oxide thin film transistor with different backchannel passivation layers, which were deposited by an ion bombardment-free process.

## Graphical abstract

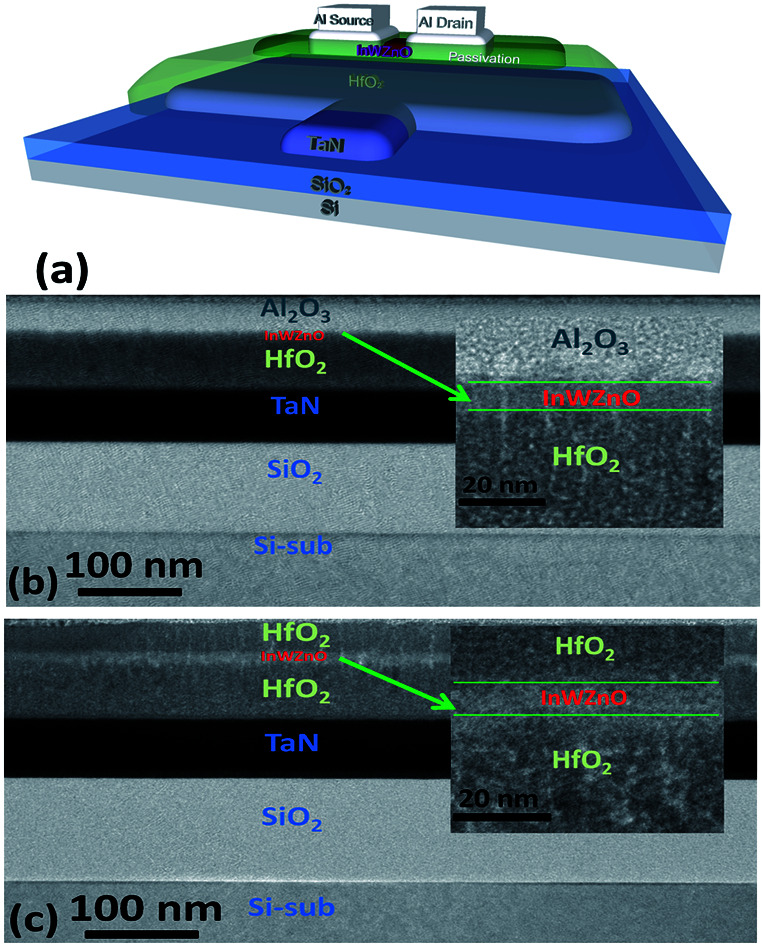

## 1. Introduction

In recent years, thin film transistors (TFTs) using transparent amorphous oxide semiconductors (TAOSs) have attracted a lot of attention from the high-resolution and large-size display industry. Higher electron mobility is the most important issue for switching devices to decrease the charging time for each pixel and the RC delay in the signal line as the panel size and the resolution requirements increase.^1^ However, there is a trade-off between a high mobility and device stability, which is caused by the density of oxygen vacancies in the channel material.^2^ Therefore, a high mobility device, which may exhibit a highly conductive channel and high concentration of oxygen vacancies, needs to overcome its reliability issues. Since Hosono *et al.* first used amorphous indium gallium zinc oxide (a-IGZO) as a channel layer,^3–7^ different types of dopant in the indium-zinc-based oxide (IZO) channel materials with good optical transmittance, like InSnZnO,^8^ InSiZnO,^9^ InHfZnO,^10^ and InZrZnO,^11^ have been widely investigated to achieve either a high carrier mobility or excellent reliability. Among these candidates, tungsten (W)-doped indium-zinc oxide (InWZnO) was proposed as an active layer material to improve bias instability due to the tungsten element's high oxygen bond dissociation energy.^12,13^ A dopant material with a high bond dissociation energy may improve the retention of oxygen atoms and lower the concentration of oxygen vacancies, and would be regarded as an excellent carrier suppressor.^14–16^ Some studies regarding the bias stability of tungsten-doped TFTs have recently been reported in detail,^17,18^ but a high mobility and good reliability TFT device with an amorphous-InWZnO channel has not been investigated yet. In addition, some research reports have indicated that oxygen vacancies should be introduced as excess carriers into the channels or backside channel interface by passivation layer deposition, which may be used as a method of enhancing the electrical mobility characteristics.^19–22^ Besides, there are some explanations regarding this mobility enhancement phenomenon, like an increase in tensile stress-induced oxygen vacancies,^19^ hydrogen generation from the precursor during the deposition process,^20^ and even generation of metallic indium by ion bombardment in a typical plasma-based deposition process.^21^ On the other hand, a TFT device with an oxygen-rich bi-layer channel structure has been proposed to suppress the excess oxygen vacancies introduced at the backside channel interface after the formation of the passivation layer by a typical plasma-based deposition process, which also improved the device mobility by lowering bulk trap-induced carrier scattering.^23^ Hence, the mechanism behind the oxygen vacancy and passivation effect has not been clearly disclosed and is worthy of further investigation.

In this work, amorphous InWZnO (a-IWZO) is used as the channel material to study the mobility enhancement effect of passivation layers with an acceptable reliability. Two types of passivation material deposited by an ion bombardment-free process, HfO_2_ and Al_2_O_3_, are selected for comparison, due to their good passivation ability to suppress gas absorption, especially H_2_O absorption. Not only that, a HfO_2_ dielectric film is chosen as a high-dielectric-constant (high-*k*) gate insulator to enhance the gate control over the conductive channel IWZO with high electron mobility as well as adjust the negative threshold voltage in a reasonable gate bias operation. Moreover, the mechanism of the passivation effect is well investigated by physical analysis and analysis of the electrical characteristics.

## 2. Experimental

TFT devices with a bottom gate staggered structure were fabricated on a doped n-type Si wafer with a 100 nm-thick thermal buffer oxide layer grown on top. The gate electrodes were then formed and patterned by sputtering a 60 nm thick TaN thin film through a shadow mask. Then, a 60 nm thick HfO_2_ layer was deposited as a gate insulator (GI) layer by electron gun (E-Gun) evaporation. After the deposition of the HfO_2_ GI layer, post deposition annealing (PDA) was performed in a thermal furnace at 300 °C for 30 min under an oxygen atmosphere, in order to improve the quality of the dielectric film and the interface between the channel and gate insulator layer. After gate region formation, a 10 nm thick InWZnO thin film was deposited as the channel layer by an RF magnetron sputtering process using a InWZnO (WO_3_ content: 4 wt%) target at room temperature. During the deposition, the flow rates of O_2_ and Ar were set to be 2 and 28 sccm, respectively. The total gas pressure in the sputter chamber was controlled to be 3 mTorr, and the optimized channel deposition conditions were applied for device fabrication in this work. Afterwards, the channel material was annealed in a thermal furnace at 300 °C under an oxygen atmosphere for 20 min. Then, the source (S) and drain (D) electrodes were formed by deposition of a 300 nm thick Al film and patterned through a shadow mask with a channel width (*W*) of 500 μm and a length (*L*) of 50 μm. Finally, two types of backchannel passivation layer (BPL) consisting of 30 nm thick Al_2_O_3_ or 30 nm thick HfO_2_ were deposited by E-Gun evaporation without plasma-induced ion bombardment effects,^24^ while a TFT device without a BPL was used as the control sample. Electrical measurements were conducted in a dark chamber at room temperature using an Agilent 4156C semiconductor parameter analyzer and a 4284A precision LCR meter. In addition, the chemical bonding states of the a-IWZO films were investigated by X-ray photoelectron spectroscopy (XPS).

## 3. Results and discussion

**Fig. 1:**
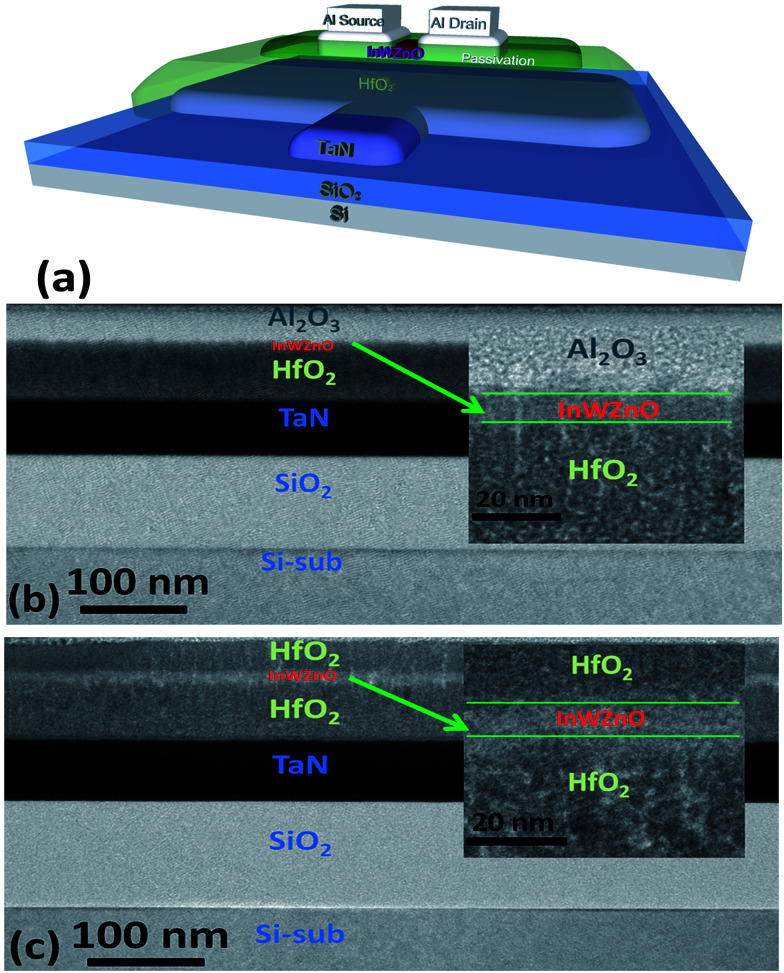
(a) A schematic device diagram of a bottom-gate TFT structure with backchannel passivation. Cross-sectional transmission electron microscopy (TEM) images of TFT device structures with (b) an Al_2_O_3_ BPL and (c) a HfO_2_ BPL.

Fig. 1(a) shows a schematic device diagram of a bottom-gate a-IWZO TFT structure with backchannel passivation. A high-*k* HfO_2_ gate insulator with a dielectric constant of 26.8 was used to increase the turn-on current (*I*_ON_) and reduce the operating voltage. In addition, the high-*k* material can effectively control the all conductive channel with a high electron mobility and carrier concentration, which is always sacrificed and abandoned due to its poor off-state characteristics in transistors with the traditional SiO_2_ gate insulator.^25^ Here, in order to achieve a qualitatively ideal a-IWZO TFT with a high mobility, the excess carrier concentration of the a-IWZO channel induced by the backchannel passivation layers needs to be suppressed by the high-*k* gate dielectric through an appropriate gate bias. Cross-sectional transmission electron microscopy (TEM) images of TFT devices with an Al_2_O_3_ BPL or a HfO_2_ BPL are shown in Fig. 1(b) and (c), respectively. The device structures including the TaN metal-gate, 60 nm thick HfO_2_ gate dielectric, 10 nm thick InWZnO channel, Al_2_O_3_ BPL and HfO_2_ BPL are all clearly observed. Previous research work has shown that the ion bombardment from Ar plasma or the sputtered particles on the backchannel of TAOSs during the RF magnetron sputtering process would break oxygen bonds and generate excess oxygen vacancies, consequently leading to an increase in the carrier concentration and unexpected surface roughness.^24,26^ In order to avoid the inevitable and randomized effects, a backchannel passivation layer was deposited on the a-IWZO layer by E-Gun evaporation in this study. A relatively distinct interface is observed between the BPL and the IWZO channel layer from the TEM image.

**Fig. 2:**
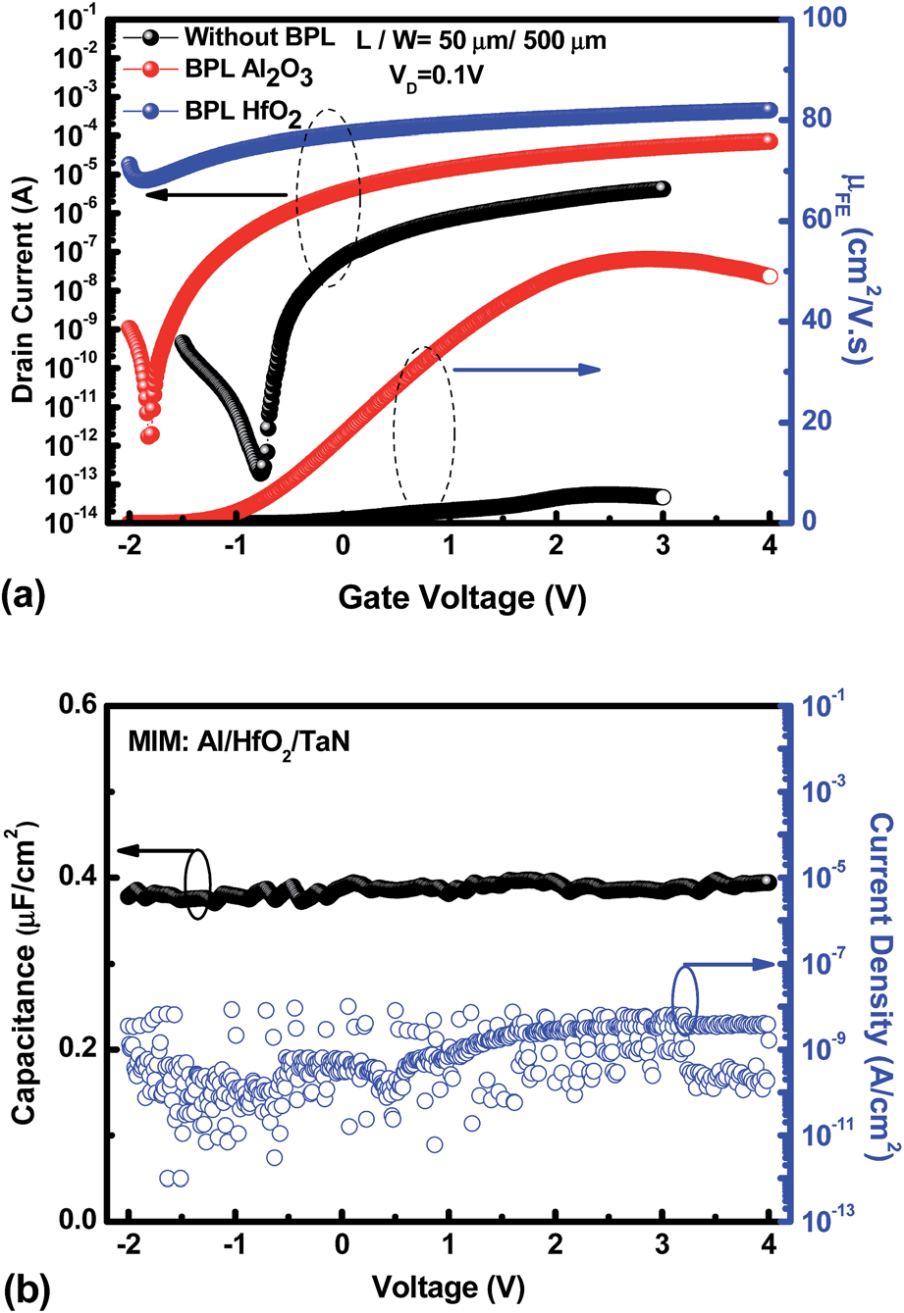
(a) The transfer characteristics and field-effect mobility of the two types of backchannel passivation TFT device structures and (b) the capacitance–voltage (*C*–*V*) and current density–voltage (*J*–*V*) characteristics of an Al/HfO_2_ GI/TaN MIM capacitor fabricated during the TFT process.

Fig. 2(a) shows the transfer characteristics and field-effect mobility (*μ*_FE_) of the a-IWZO TFT devices with an Al_2_O_3_ BPL and a HfO_2_ BPL, and the control device without a BPL, respectively. The field-effect mobility was extracted from the transconductance at a drain current in the linear region (*V*_D_ = 0.1 V). The subthreshold swing (SS) was calculated from the gate-to-source voltage (*V*_GS_), which was determined by an increase in drain current (*I*_D_) from 10^10^ to 10^8^ A. The threshold voltage (*V*_TH_) was defined as the *V*_GS_ at a normalized drain current of 10 nA. The control sample shows an acceptable performance with a peak *μ*_FE_ of 5.67 cm^2^ V^−1^ s^−1^, an *I*_ON_/*I*_OFF_ of 2.21 × 10^7^, a SS of 98.1 mV dec^−1^, and a *V*_TH_ of −0.34 V. In contrast to the control one, the sample with an Al_2_O_3_ BPL exhibits a high peak *μ*_FE_ value (52.5 cm^2^ V^−1^ s^−1^), a relatively large *I*_ON_/*I*_OFF_ ratio (1 × 10^7^), a small SS of 107.7 mV dec^−1^ and a low *V*_TH_ of −1.43 V, while the sample with a HfO_2_ BPL shows a highly conductive channel which cannot be depleted by a reasonable gate bias in these process conditions. In this work, all samples show a similar surface quality at the interface of the front channel between the IWZO active layer and the HfO_2_ GI layer. This suggests that the passivation layer material is the only factor that can influence the density of oxygen vacancies in the IWZO channel without considering the hydrogen generation or ion bombardment from the plasma-based passivation deposition process. The neutral oxygen vacancies are easily excited into the charged state in the form of donor-like oxygen vacancies (V_O_^2+^).^27^ As a result, the increase in carrier concentration induced by the BPL process may lead to a 10 times enhancement in the peak value of *μ*_FE_ and a slightly negative shift of *V*_TH_ for the TFT with Al_2_O_3_. Fig. 2(b) shows the capacitance–voltage (*C*–*V*) and current density–voltage (*J*–*V*) characteristics of an Al/high-*k* dielectric GI/TaN MIM capacitor fabricated during the TFT process. A capacitance density of 0.395 μF cm^−2^ was measured at a frequency of 100 KHz, while the leakage current was lower than 5 × 10^−8^ A cm^−2^ even at 4 V. The quality of the high-*k* GI is controlled by the optimization of the deposition rate and PDA conditions. In addition, the on-state current of the a-IWZO TFT can be further improved at a relatively low operation voltage by such a high value of the oxide capacitance.

**Fig. 3:**
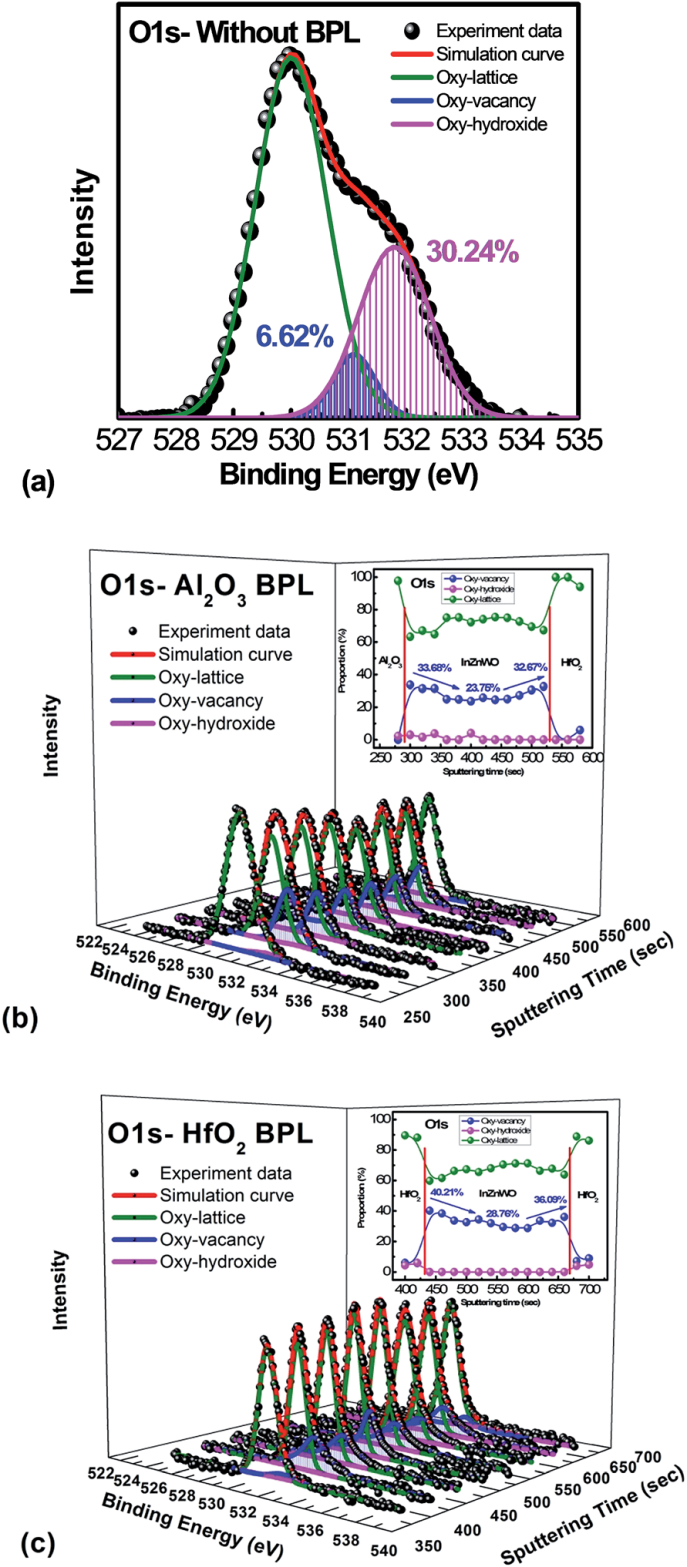
(a) Analysis of the XPS O 1s spectrum on the backchannel surface of an InWZnO thin film without a passivation layer. The depth distribution analysis profile of the XPS O 1s spectrum for the a-InWZnO films with (b) an Al_2_O_3_ BPL and (c) a HfO_2_ BPL; the inset image summarizes the proportion of oxygen binding states.

To further investigate the physical mechanism for the mobility improvement caused by the passivation layer deposition process, material analysis was performed. The XPS spectrum of the O 1s signal was applied to examine the oxygen binding states at the backchannel surface of the InWZnO thin film. As shown in Fig. 3(a) for the IWZO channel without a passivation layer, the O 1s peak can be fitted by three nearly Gaussian distributions, approximately centered at an energy of 529.8, 531.1 and 531.8 eV, respectively.^23^

**Fig. 4:**
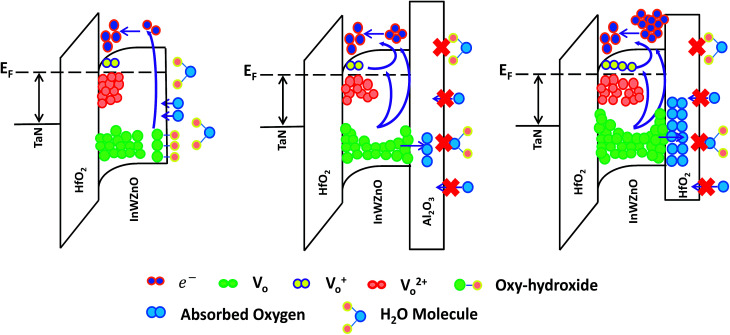
Schematic band diagrams of the a-IWZO TFT with or without a BPL, which conceptually depict the mechanism of mobility enhancement caused by the increase in carrier concentration.

For the lowest binding energy peak located at 529.8 eV, the signal is attributed to oxygen-lattice bonds, which are related to the O^2−^ ions combined with the W, In, and Zn atoms in the a-InWZnO compound system. Then, for the highest binding energy peak located at 531.8 eV, the signal is attributed to oxygen–hydroxide bonds. This peak is associated with the loosely bound oxygen at the surface of the InWZnO film, which can be terminated with a specific chemisorbed oxygen, such as CO_2_, absorbed H_2_O, or absorbed O_2_. For the middle binding energy component located at 531.1 eV, the signal is attributed to oxygen-vacancy bonds. The O_V_ peak is attributed to O^2−^ ions that are in an oxygen deficient region of the a-InWZnO matrix. It is important to notice that there is a significant amount of oxygen–hydroxide bond signal (about 30.24%) for the sample without passivation, while a low proportion of oxygen-vacancy bonds (about 6.62%) was also observed. On the other hand, Fig. 3(b) and (c) show the depth distribution profiles of the XPS O 1s spectra for the a-InWZnO films with (b) an Al_2_O_3_ BPL or (c) a HfO_2_ BPL. The XPS results regarding the depth distribution of the three types of oxygen bond for the a-InWZnO channel material are summarized in the inset of Fig. 3(b) and (c), respectively. According to the XPS results, the TFT with a HfO_2_ BPL shows a higher proportion of donor-like oxygen-vacancy bonds (28.76∼40.21%) than the Al_2_O_3_ BPL TFT (23.76∼33.67%). This may be the reason for its high carrier concentration, which may cause the device to be identified as a nonlinear gate controlled resistor, rather than a transistor. On the other hand, compared with the TFT without passivation, both of the TFTs with a BPL show a significant decrease in the signal of the oxygen-hydroxide peak (lower than 2%). This indicates that BPL materials can block hydrogen diffusion effectively and show a good passivation ability to suppress gas absorption, especially H_2_O absorption. In order to conceptually depict the mechanism of the mobility enhancement caused by the increase in carrier concentration, schematic band diagrams of the a-IWZO TFT with or without a BPL are shown in Fig. 4. In general, hydrogen will incorporate into the a-InWZnO thin film due to hydrogen diffusion in the backchannel. Afterwards, the hydrogen will react with oxygen and generate oxygen-hydroxide bonds and excess carriers.^23^ In fact, the excess carriers also lead to an improvement in mobility, but the enhancement is not stable and it is easily degraded by the absorbed oxygen under ambient atmosphere. On the other hand, both of the BPL TFTs with a similar and small amount of oxygen–hydroxide bond signal still show an increase in channel conductivity from Fig. 2(a), 3(b) and (c). The reason for this may be the increase in the intensity of the signal of oxygen-vacancy bonds acting as donor-like states for both BPL TFTs, as shown in Fig. 4. According to the percolation conduction model for AOS-based TFTs, the conduction band minimum (CBM) of the channel material is formed by the unoccupied s orbitals.^28^ The increase in donor-like oxygen vacancies may enhance the carrier mobility with increasing carrier concentration. However, the HfO_2_ BPL may induce such a high carrier concentration that a reasonable gate bias cannot turn the TFT device off easily in this study. The difference in channel conductivity between the two BPL samples can be attributed to the difference in Gibbs free energy between HfO_2_ (−260.1 kcal mol^−1^) and Al_2_O_3_ (−377.9 kcal mol^−1^), which may influence their ability to react with oxygen in the channel.^29^ Besides, the oxygen bond dissociation energies of Al–O and Hf–O are 791 kJ mol^−1^ and 512 kJ mol^−1^, respectively, which are higher than that of In–O (360 kJ mol^−1^).^16^ This means that the oxygen in the a-IWZO channel can be desorbed easily by the backchannel passivation material, which may also cause an increase in oxygen vacancies with donor-like state behavior in the channel. Furthermore, there is a slight increase in oxygen-vacancy bonds at both the BPL/a-InWZnO and a-InWZnO/HfO_2_ GI interfaces. This can be attributed to more oxygen precipitation occurring at the back side and front side of the channel layer.

**Fig. 5:**
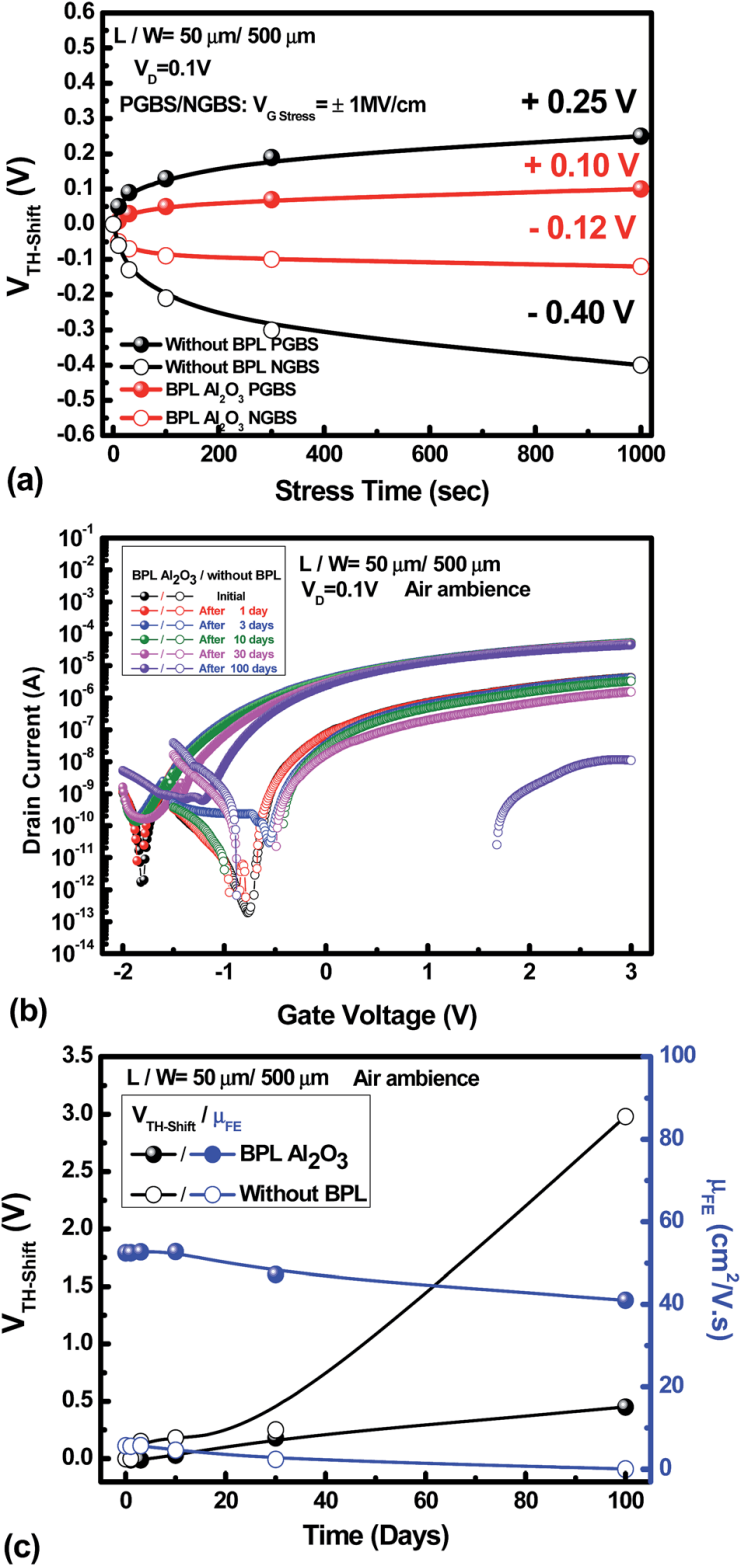
(a) The values of threshold voltage shift (*V*_TH-shift_) *versus* the stress time under PGBS or NGBS for the a-IWZO TFT with an Al_2_O_3_ BPL and the one without passivation. (b) The transfer characteristics of the a-IWZO TFT with an Al_2_O_3_ BPL and the one without passivation exposed to the ambient air for 100 days. (c) The *V*_TH-shift_ values and field-effect mobility degradation *versus* the exposure time under ambient air.

AOS materials have been reported to suffer from reliability degradation, and research has indicated that the instability can be related to the interaction of the backchannel surface and the ambient atmosphere.^21^ Many attempts have been made to improve the reliability issue. However, the mobility is sacrificed in these cases. Table 1 shows a comparison of the IWZO TFT device performance with the performance of devices with similar channel materials reported in recent years. It exhibits the obvious mobility enhancement of the IWZO TFT device that is achieved in this work. On the other hand, it also suggests that the reliability characteristics need to be maintained and investigated. Fig. 5(a) shows the values of threshold voltage shift (*V*_TH-shift_) *versus* the stress time under positive gate bias stress (PGBS) or negative gate bias stress (NGBS) for the a-IWZO TFT with an Al_2_O_3_ BPL and the one without a passivation layer. The stress conditions are as follows: *V*_G stress_ = +6 V (*E*_G stress_ = +1 MV cm^−1^) for PGBS, *V*_G stress_ = −6 V (*E*_G stress_ = −1 MV cm^−1^) and *V*_D_ is fixed at 0.1 V for 1000 s. It reveals that the reliability characteristics of high-stability channel material IWZO TFT devices can be further improved by an Al_2_O_3_ BPL under PGBS and NGBS as compared to the ones without a BPL. In addition, Fig. 5(b) shows the transfer characteristics of the a-IWZO TFT with an Al_2_O_3_ BPL and the one without a BPL exposed to the ambient air for over three months. After 100 days, the a-IWZO TFT with an Al_2_O_3_ BPL still shows a remarkable lifetime and a high mobility, while the TFT without a BPL shows obvious degradation during 30 days. This indicates that the Al_2_O_3_ BPL can effectively keep the oxygen concentration stable in the a-IWZO channel layer and protect against the influence of the ambient air on the carrier concentration. Fig. 5(c) summarizes the shift in *V*_TH_ value and field-effect mobility degradation *versus* the exposure time in the ambient atmosphere. It is found that the a-IWZO TFT device without a BPL shows a positive threshold voltage shift of 0.25 V after 30 days and 2.98 V after 100 days. This is much larger than that of the TFT device with an Al_2_O_3_ BPL, which is 0.18 V after 30 days and 0.45 V after 100 days.

**Table 1:** Comparison of the device performance with the performance of devices with similar high stability channel materials reported in recent years

GI/channel	*μ*_FE_ (cm^2^ V^−1^ s^−1^)	*V*_TH_ (V)	SS (V dec^−1^)	*I*_ON_/*I*_OFF_
Al_2_O_3_/IWZO^12^	11.1	4	0.31	1 × 10^7^
SiO_2_/IWZO^13^	19.57	−0.4	0.14	∼10^7^
SiO_2_/IWO^17^	26.5	−2.5	0.5	∼10^7^
SiO_2_/IWZO^18^	40	−1.6	NA	1.8 × 10^11^
This work	52.5	−1.43	0.1077	1 × 10^7^

## 4. Conclusions

In summary, a low temperature amorphous InWZnO TFT with a high-*k* GI and Al_2_O_3_ BPL is proposed for achieving good subthreshold swing, acceptable *I*_ON_/*I*_OFF_ ratio and a 10 times enhancement in field-effect mobility in this study. The mechanism of the mobility enhancement effects is ascribed to the generation of donor-like oxygen vacancies at the backchannel by the suitable passivation material, which was deposited by an ion bombardment-free process and exhibited a good passivation ability to suppress H_2_O absorption. Analysis of the XPS O 1s depth distribution profile has been used to confirm this inference. In the case of an Al_2_O_3_ layer, the Gibbs free energy of the reactions with oxygen is the most negative among the BPL materials studied in this work. Also, the large oxygen bond dissociation energy for Al_2_O_3_ can effectively desorb oxygen in the backchannel layer. Therefore, the integration of a HfO_2_ GI and an a-IWZO TFT with an Al_2_O_3_ BPL can result in superior electrical characteristics and a remarkable stability compared with other passivation materials. This study proposes an approach for synthesizing AOS-based TFTs to achieve a high field-effect mobility without decreasing the device reliability.

## Conflicts of interest

There are no conflicts to declare.

## Supplementary Material

## References

[cit1] Fortunato E., Barquinha P., Martins R. (2012). Adv. Mater..

[cit2] Kizu T., Aikawa S., Mitoma N., Shimizu M., Gao X., Lin M. F., Nabatame T., Tsukagoshi K. (2014). Appl. Phys. Lett..

[cit3] Nomura K., Ohta H., Takagi A., Kamiya T., Hirano M., Hosono H. (2004). Nature.

[cit4] Kamiya T., Nomura K., Hosono H. (2010). Sci. Technol. Adv. Mater..

[cit5] Nomura K., Kamiya T., Hosono H. (2012). Thin Solid Films.

[cit6] Tiwari N., Chauhan R. N., Liu P. T., Shieh H. P. D. (2015). RSC Adv..

[cit7] Tiwari N., Chauhan R. N., Liu P. T., Shieh H. P. D. (2016). RSC Adv..

[cit8] Liu P. T., Chang C. H., Fuh C. S. (2016). RSC Adv..

[cit9] Chong E., Chun Y. S., Lee S. Y. (2010). Appl. Phys. Lett..

[cit10] Kim C. J., Kim S., Lee J. H., Park J. S., Kim S., Park J., Lee E., Lee J., Park Y., Kim J. H., Shin S. T., Chung U. I. (2009). Appl. Phys. Lett..

[cit11] Park J. S., Kim K. S., Park Y. G., Mo Y. G., Kim H. D., Jeong J. K. (2009). Adv. Mater..

[cit12] Li H., Qu M., Zhang Q. (2013). IEEE Electron Device Lett..

[cit13] Park H. W., Park K., Kwon J. Y., Choi D., Chung K. B. (2017). IEEE Trans. Electron Devices.

[cit14] Aikawa S., Nabatame T., Tsukagoshi K. (2013). Appl. Phys. Lett..

[cit15] Aikawa S., Mitoma N., Kizu T., Nabatame T., Tsukagoshi K. (2015). Appl. Phys. Lett..

[cit16] Dean J. A. , Lange's handbook of chemistry, 1998, vol. 4, p. 41

[cit17] Yang Z., Meng T., Zhang Q., Shieh H. P. D. (2016). IEEE Electron Device Lett..

[cit18] Kizu T., Mitoma N., Miyanaga M., Awata H., Nabatame T., Tsukagoshi K. (2015). J. Appl. Phys..

[cit19] Liu S. E., Yu M. J., Lin C. Y., Ho G. T., Cheng C. C., Lai C. M., Lin C. J., King Y. C., Yeh Y. H. (2011). IEEE Electron Device Lett..

[cit20] Nguyen T. T. T., Aventurier B., Terlier T., Barnes J. P., Templier F. (2015). J. Disp. Technol..

[cit21] Hu S., Lu K., Ning H., Zheng Z., Zhang H., Fang Z., Yao R., Xu M., Wang L., Lan L., Peng J., Lu X. (2017). IEEE Electron Device Lett..

[cit22] Shih C. W., Chin A. (2017). Sci. Rep..

[cit23] Liu P. T., Chang C. H., Chang C. J. (2016). Appl. Phys. Lett..

[cit24] Olziersky A., Barquinha P., Vilà A., Pereira L., Gonçalves G., Fortunato E., Martins R., Morante J. R. (2010). J. Appl. Phys..

[cit25] Park J. S., Kim H., Kim I. D. (2014). J. Electroceram..

[cit26] Chen T. C., Chang T. C., Hsieh T. Y., Tsai C. T., Chen S. C., Lin C. S., Hung M. C., Tu C. H., Chang J. J., Chen P. L. (2010). Appl. Phys. Lett..

[cit27] Janotti A., Van de Walle C. G. (2005). Appl. Phys. Lett..

[cit28] Lee S., Ghaffarzadeh K., Nathan A., Robertson J., Jeon S., Kim C., Song H., Chung U. (2011). Appl. Phys. Lett..

[cit29] Salihoglu O., Tansel T., Hostut M., Ergun Y., Aydinli A. (2016). Proc. SPIE.

